# Colorimetric Detection of Uranyl Using a Litmus Test

**DOI:** 10.3389/fchem.2018.00332

**Published:** 2018-08-09

**Authors:** Sepehr Manochehry, Erin M. McConnell, Kha Q. Tram, Joseph Macri, Yingfu Li

**Affiliations:** ^1^Department of Biochemistry and Biomedical Sciences, McMaster University Hamilton, ON, Canada; ^2^Department of Chemistry and Chemical Biology, McMaster University Hamilton, ON, Canada; ^3^Department of Pathology and Molecular Medicine, McMaster University Hamilton, ON, Canada; ^4^Hamilton Regional Laboratory Medicine Program Hamilton, ON, Canada

**Keywords:** biosensor, DNAzyme, colorimetric detection, litmus test, uranyl

## Abstract

Ingestion of water containing toxic contaminants above levels deemed safe for human consumption can occur unknowingly since numerous common contaminants in drinking water are colorless and odorless. Uranyl is particularly problematic as it has been found at dangerous levels in sources of drinking water. Detection of this heavy metal-ion species in drinking water currently requires sending a sample to a laboratory where trained personnel use equipment to perform the analysis and turn-around times can be long. A pH-responsive colorimetric biosensor was developed to enable detection of uranyl in water which coupled the uranyl-specific 39E DNAzyme as a recognition element, and an enzyme capable of producing a pH change as the reporter element. The rapid colorimetric assay presented herein can detect uranyl in lake and well water at concentrations relevant for environmental monitoring, as demonstrated by the detection of uranyl at levels below the limits set for drinking water by major regulatory agencies including the World Health Organization (30 μg/L). This simple and inexpensive DNAzyme-based assay enabled equipment-free visual detection of 15 μg/L uranyl, using both solution-based and paper-based pH-dependent visualization strategies.

## Introduction

Numerous contaminants commonly found in drinking water can go undetected due to their lack of distinguishable appearance or taste. One such contaminant is the radioactive element uranium, a heavy metal which occurs most commonly in aqueous solutions as the uranyl dication, UO${}_{2}^{2+}$ (Cothern and Lappenbusch, 1983; Burkart et al., 2005; World Health Organization, 2012). Exposure to uranyl can result in negative health effects, due to both the element's chemical toxicity and its radioactive properties. Examples of these negative effects include: acute kidney failure (Lu and Zhao, 1990; Zamora et al., 1998), developmental disabilities (Shields et al., 1992; Domingo, 2001), reproductive disabilities (Paternain et al., 1989; Domingo, 2001), and DNA damage (Zaire et al., 1996).

A particularly dangerous source of exposure to uranium is contaminated drinking water, consumption of which exposes the cells of the human body to alpha and gamma emissions at a very short proximity (Cothern and Lappenbusch, 1983). Major sources of uranium in ground water include leachate from natural deposits (Weir, 2004), mill tailings (Dreesen et al., 1982), emissions from the nuclear industry (Anirudhan et al., 2010), and combustion products from fossil fuels (Tadmor, 1986). As with other heavy metal contaminants such as lead, the substantial health risks associated with uranyl ingestion have prompted governmental agencies, such as Canada's Federal-Provincial-Territorial Committee on Drinking Water (CDW), the United States Environmental Protection Agency (EPA) and the World Health Organization (WHO), to recommend periodic testing of private wells. This testing is pertinent since these regulatory bodies do not monitor the water from private wells. When the onus falls on private well owners with limited resources, contaminants can go unnoticed at levels above those determined safe for human consumption by regulatory guidelines (Betcher et al., 1988; Health Canada, 2001; US Environmental Protection Agency, 2001; World Health Organization, 2012).

Traditional approaches to detection of uranyl have utilized various physical and chemical techniques, including solid fluorimetry (Krieger and Whittaker, 1980), inductively coupled plasma-mass spectrometry (Boomer and Powell, 1987), inductively coupled plasma-atomic emission spectrometry (Huff and Bowers, 1990), radio spectrometry (Holzbecher and Ryan, 1980), stripping voltammetry (Mlakar and Branica, 1989), atomic adsorption spectrometry (Abbasi, 1989), and phosphorimetry (Kaminski et al., 1981; Brina and Miller, 1992). The most commonly used method for the detection of uranyl in water is solid fluorimetry (Krieger and Whittaker, 1980), a method which requires tedious sample preparation, and can be complicated by interference from other metals. Another common method is inductively coupled plasma mass spectrometry (Boomer and Powell, 1987). Both methods must be performed in a specialized laboratory by highly trained personnel, have long turn-around times, and are expensive. These factors can be a significant barrier to maintaining safe drinking water.

Globally, millions of people rely on ground-water as their drinking supply, therefore, there is a direct need for the development of a simple, quick, and reliable method of testing water sources for this dangerous contaminant. One strategy for the detection of environmental contaminants is the development of sensors based on RNA-cleaving DNAzymes (RCDs). RCDs are synthetic single-stranded DNA molecules that are selected *in vitro*, from a random-sequence DNA pool, to perform catalytic cleavage of a phosphodiester bond in an RNA-containing sequence (Breaker and Joyce, 1994). For example, diverse RCDs have been derived to catalyze the cleavage of the phosphodiester bond at the location of the lone ribonucleotide embedded in a DNA chain (Silverman, 2005; Schlosser and Li, 2009; Liu et al., 2017; McGhee et al., 2017; Zhou et al., 2017; Morrison et al., 2018). Many of the reported RCDs are specially engineered such that their catalytic activity is dependent on another specific molecule, and therefore, these RCDs can be used as the recognition element for the design of a target-specific biosensor (Liu et al., 2007; Ali et al., 2011; Tram et al., 2014; McGhee et al., 2017). One such example is 39E, a uranyl-specific RCD derived by the Lu group in 2007. This DNAzyme exhibited a limit of detection of 11 parts per trillion (45 pM), and greater than 1 million-fold selectivity over many other metal ions (Liu et al., 2007). The DNAzyme's sensitivity and selectivity rivals the detection capabilities of the more commonly used analytical instruments. Since its isolation, the 39E DNAzyme has been used in a broad array of biosensing strategies where the generated signal was measured using fluorescence (Liu et al., 2007; He and Lu, 2011; Xiao et al., 2015; Zhang et al., 2015; Cao et al., 2016; Zhou et al., 2016b), electrochemistry (Xiang and Lu, 2011; Tang et al., 2013; Yun et al., 2016a,b), resonance light scattering (Zhou et al., 2013), and surface-enhanced Raman spectroscopy (Gwak et al., 2016). Furthermore, 39E has been utilized for imaging of uranyl in live cells (Wu et al., 2013), and has been coupled to a contrast agent for magnetic resonance imaging (Xu et al., 2013). 39E has also been used in a variety of colorimetric detection assays, which employed both enzymatic (e.g., peroxidase) and non-enzymatic [e.g., gold nanoparticle (AuNP)] signal generation methods (Lee et al., 2008; Luo et al., 2012; Huang et al., 2016; Zhang et al., 2016, 2017; Cheng et al., 2017; Yun et al., 2018). These enzymatic approaches utilize peroxidase, or peroxidase-mimetic components, while the non-enzymatic AuNP-based signal generation strategies largely rely on aggregation-dependent differences in AuNP absorbance to produce a visible signal. By utilizing the powerful 39E DNAzyme, detection of uranyl in complex samples such as river water has been previously demonstrated using a colorimetric signal generation approach that relies on horseradish peroxidase (HRP)-assisted catalytic oxidation of 3,3′,5,5′ tetramethylbenzidine (TMB) to produce a change from clear to blue (Zhang et al., 2016, 2017; Cheng et al., 2017).

**Figure 1:**
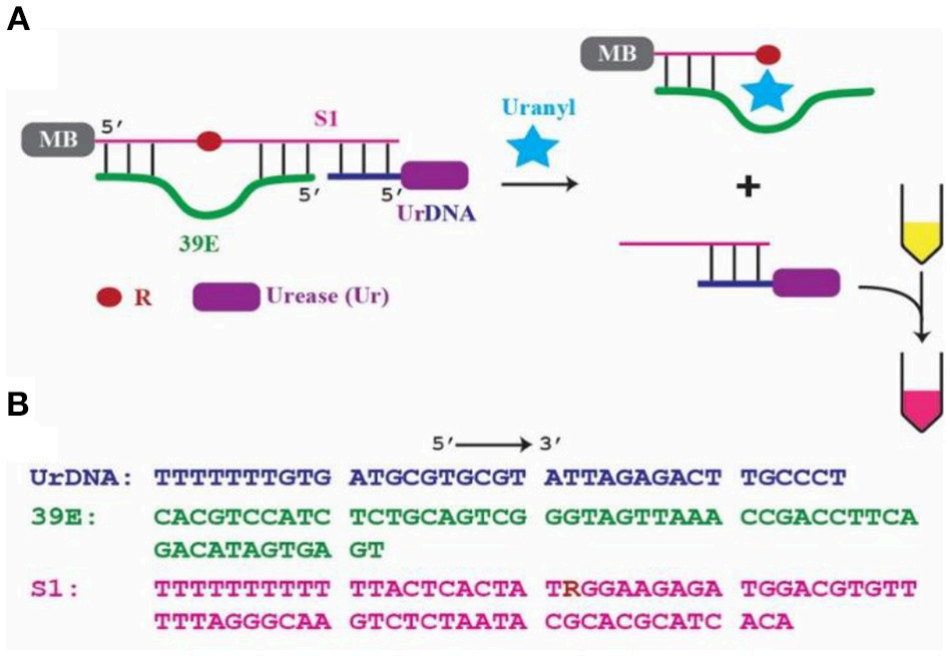
39E-based colorimetric detection of uranyl. **(A)** Schematic of the 39E/urease construct with covalently linked biotin placed at the 5′ of the substrate S1 complexed with streptavidin coated magnetic beads, indicated by MB, and urease-linked DNA shown as UrDNA. **(B)** Sequences of 39E-UrDNA system, composed of urease-linked DNA (UrDNA), uranyl-specific DNAzyme (39E), substrate (S1) with R indicating adenine ribonucleotide junction cleaved by the DNAzyme.

We report a colorimetric detection method based on the 39E DNAzyme and the classic litmus test. The resulting biosensor, shown schematically in Figure 1, is simple and modular, as it is composed of a DNAzyme which detects the target, and a pH-increasing enzyme which indirectly produces an optical signal that can be visualized using litmus dyes or litmus paper. The litmus test for pH is a well-established colorimetric sensor that has been widely used for many years, and as a result, reagents like litmus dyes and pH paper are inexpensive, commercially available, easy to use, and easy to interpret. These factors are critical to the effectiveness of a biosensor, thus it is important to further the research into colorimetric approaches that take advantage of the litmus test in applications where on-site biosensing may be valuable.

This work is based on a system previously demonstrated by our group (Tram et al., 2014), where the DNAzyme component was specific to *Escherichia coli* (*E. coli*). In this system, the DNAzyme (39E, green) was hybridized to its substrate (S1, pink). This complex was immobilized on the surface of a magnetic bead (MB) through the 5′-end of the substrate molecule. Importantly, a complementary oligonucleotide-urease conjugate (UrDNA, purple) was hybridized to the 3′-end of the substrate molecule. Here the DNAzyme responsible for detecting uranyl and the enzyme that facilitates the conversion of this recognition event into a visible signal interact non-covalently through hybridization of the complementary components. Following uranyl-induced cleavage of the substrate, the supernatant containing the cleaved portion which remained hybridized with UrDNA was transferred to another tube in which urea and phenol red (a pH indicator) were added. The urease enzyme catalyzed the hydrolysis of urea, yielding carbon dioxide and ammonia. This anhydrous ammonia (NH_3_) readily reacts with water to produce free ammonium ions (NH${}_{4}^{+}$) and hydroxide ions (OH^−^). The resultant increase in solution pH was indicated by the vibrant color change of the phenol red (Sumner and Hand, 1929; Dunn et al., 1990; Karplus et al., 1997). The inspiration for this sensor came from the inexpensive and commonly employed litmus assay. Coupling a molecular cleavage event to a change in solution pH allowed for highly sensitive colorimetric detection using reagents that are commercially available and inexpensive (e.g., urease, phenol red, pH paper).

Though this colorimetric DNAzyme-based biosensing approach is simple and transferable to numerous DNAzymes, the *E. coli* DNAzyme is the only example that has been demonstrated to date. We chose to examine the uranyl DNAzyme, 39E, in an effort to develop a biosensor capable of detecting this important drinking water contaminant in well water and lake water. Considering the importance of monitoring levels of this toxic heavy metal in drinking water, we set out to demonstrate the equipment-free colorimetric biosensing of uranyl at levels below regulatory limits set by Canada, the US, and the WHO, both in solution using phenol red, and by using commercially available pH paper.

## Materials and methods

### Chemicals and reagents

Oligonucleotides were purchased from IDT DNA Technologies (Coralville, IA, USA). The oligonucleotide component of UrDNA (5′-Amino modifier C6-TTTTT TTGTG ATGCG TGCGT ATAAG AGACT TGCCC T-3′), the DNAzyme 39E (5′-CACGT CCATC TCTGC AGTCG GGTAG TTAAA CCGAC CTTCA GACAT AGTGA GT-3′), and its modified substrate S1 (5′-Biotin-TTTTT TTTTT TTACT CACTA TRGGA AGAGA TGGAC GTGTT TTTAG GGCAA GTCTC TAATA CGCAC GCATC ACA-3′; R = adenine ribonucleotide), were PAGE purified prior to use as previously described (Tram et al., 2014). BioMag Streptavidin, nuclease-free, magnetic beads were purchased from Bangs Laboratories (Fischers, IN, USA). Uranyl dilutions were prepared in 10 × stock concentrations corresponding to half of the maximum acceptable concentration (MAC) specified by guidelines provided by the WHO in addition to that of federal regulatory agencies in the United States, and Canada. The WHO and US share a value of 30 μg/L, while the Canadian standard is set slightly lower, at 20 μg/L (Health Canada, 2001; US Environmental Protection Agency, 2001; World Health Organization, 2012). Thus 100 and 150 μg/L stocks of uranyl were prepared, using uranyl acetate dihydrate from Ted Pella (Redding, CA, USA). Lead acetate and mercury chloride were used to prepare 10 × stock with concentrations corresponding to MAC levels set by the WHO, at 100 and 60 μg/L, respectively (Gorchev and Ozolins, 2011). No MAC is set for magnesium therefore a 10 × stock was prepared at 2000 μg/L.

For paper-based tests, Hydrion MicroFine 5.5–8.0 pH paper was used, which was purchased from MicroEssential Laboratories (Brooklyn, NY, USA). All other components were purchased from Sigma Aldrich (Oakville, Canada).

Distilled Milli-Q water was used for all experiments unless otherwise stated, from hereon referred to as ddH_2_O. Well water and lake water samples were spiked with uranyl to reach a final concentration of half the MAC specified by the WHO and the United States' EPA. Well water was obtained through a source from the Ontario Ground Water Association (OGWA), and lake water was obtained from Lake Ontario at the Niagara region. To prepare spiked environmental water samples for testing, raw lake water and well water samples were filtered using a sterile syringe and a 0.22-μm nitrocellulose filter, and uranyl was added to prepare corresponding 10 × stocks. The inclusion of a filtration step was intended to remove potential large particles, such as sand, plant matter, bacteria, that may interfere with the test. Importantly, this operation did not alter the metal ion compositions and their concentrations by element analysis to be detailed below.

### Apparatus and instruments

Routine electrolytes (Ca, Cl, K, HCO3, Mg, Na) were measured on the Abbott Architect ci16200 integrated diagnostic platform (Lake Bluff, Illinois, USA) according to manufacturer's guidelines. Ion selective electrodes were used to measure Na, K, and Cl. Ca was measured using a colorimetric Arsenazo-III dye reaction while Mg and HCO3 were determined based on an enzymatic reaction utilizing isocitrate dehydrogenase and malate dehydrogenase respectively. Tuning reagents and internal standards are from Agilent Technologies (Tokyo, Japan). All trace elements standards were purchased from Sigma Aldrich (Oakville, Canada). Trace element measurements were conducted using the Agilent 8800 triple-quadrupole ICP-MS instrument (ICP-QQQ/Agilent Technologies, Tokyo, Japan) using a modification of a previously described protocol (Macedo et al., 2017). Briefly, calibration standards and water samples were prepared by a 3-fold dilution in 0.5% NH_4_OH (wt) containing 2% (wt) isopropanol, 0.025% (wt) EDTA, 0.025% (wt) Triton X-100 as well as internal standards (Bi, Ge, In, Li, Lu, Rh, Sc, and Tb) with an individual concentration of 420 μg/L. A total of seven calibration standards were prepared for trace element quantitation. Analysis was conducted using with either He, H, or O as a reaction gas and an integration time of 0.5 s.

Single-point absorbance readings were measured at 557 nm using a Varian Cary 100 spectrophotometer (Darmstadt, Germany), with 1 cm path length quartz cuvettes from BioBasic Inc. (Markham, Canada). Spectrophotometric measurements were used to determine the limit of detection (LOD), based on signal greater than three times of background variation. The linear range assessed for determination of LOD was set to correspond to less than 20% of maximal signal generated. Photographs of color changes were captured using a Canon PowerShot G11 digital camera (Tokyo, Japan), under manual configuration with 100 ISO and macro activated. Photoshop version 15.0.0 was used to correct both white-balance and decrease brightness to −20 for all colorimetric photos.

### Assembly of the 39E and urease containing DNA complex 39E-UrDNA

All tubes were pre-washed with binding buffer (0.5 M NaCl, 20 mM Tris-HCl, 1 mM MgCl_2_, 0.01% v/v Tween20, pH 8). 100 μL of streptavidin-coated magnetic beads were washed with 150 μL of binding buffer. The wash was removed using magnetic separation, and the beads were resuspended in 90 μL of binding buffer prior to addition of 10 μL of 20 μM biotinylated substrate S1. The suspension was incubated at room temperature for 30 min. The magnetic beads were then washed twice with 150 μL of binding buffer and then resuspended in 170 μL of binding buffer. 30 μL of 20 μM of 39E was then added to the suspension, followed by heating at 65°C for 2 min and cooling to room temperature over 10 min. Then 20 pmol of urease linked DNA (UrDNA) synthesized in accordance with a previously published protocol was added and incubated at 37°C for 10 min (Tram et al., 2014). The suspension was then allowed to cool for 15 min at room temperature before washing with 100 μL of binding buffer once, and then performing a second wash with reaction buffer (0.3 M NaCl, 5 mM MES, 0.01% v/v Tween 20), followed by resuspension in 100 μL of reaction buffer.

### DNAzyme-based colorimetric litmus assay

2.5 μL of 10 × stocks of uranyl, at 15, 100, and 150 μg/L, were incubated with 22.5 μL of the aforementioned biosensor complex in reaction buffer at room temperature for up to 60 min. Minimum cleavage time was assessed using the protocol outlined by Tram et (Tram et al., 2014). When the reaction was complete, 150 μL of ddH2O were added to the suspension which was then placed on a magnetic rack to allow isolation of the supernatant. 20 μL of supernatant were transferred to another microcentrifuge tube, combined with 2.5 μL 0.04% phenol red, and 25 μL of urea substrate solution (2 M NaCl, 60 mM MgCl2, 50 mM urea, 0.1 mM acetic acid, pH 5.0). Similarly, for absorbance measurements using the spectrophotometer, 80 μL of supernatant was transferred to a quartz cuvette, combined with 10 μL of 0.04% phenol, and 100 μL of the urea substrate solution. Curve fitting was performed using the four-parameter sigmoid model on SigmaPlot 12.0 for Windows (Chicago, IL, USA). Phenol red has previously been shown to be the optimal choice of indicator since it is vibrantly yellow at near neutral pH and transitions to bright purple at basic pH values above 8.2 (Rovati et al., 2012; Tram et al., 2014). This translates to a clear contrast between test and control samples that enables rapid visual determination of the presence of target.

### Paper-based litmus assay

Tests with pH paper used these same conditions as indicated above, with the exception that the solution did not contain phenol red. Instead, pH paper was used to visualize the pH-dependent colorimetric response. Following incubation with varying concentrations of uranyl, the magnetic beads were separated; 20 μL of the supernatant was transferred to another microcentrifuge tube, and combined with 25 μL of the urea substrate solution. Following the incubation in the urea solution, an aliquot was removed, deposited onto pH paper and incubated for 1 min to allow for the pH paper to change color prior to photo capture.

## Results and discussion

### Design of the 39E and urease conjugate (39E-UrDNA)

The 39E-UrDNA system employs an enzyme-based signal generation strategy. In this simple test, the water sample was added to a solution of magnetic beads with surface immobilized 39E-UrDNA. After a brief incubation period, the tube was placed adjacent to a magnet, and the solution containing the product of the uranyl-induced cleavage reaction by the 39E DNAzyme was transferred into a new microcentrifuge tube. This second tube contained the necessary reagents for the litmus reaction (urea and phenol red), thereby enabling visualization of the urease-catalyzed hydrolysis of urea. In the absence of uranyl-induced cleavage, the UrDNA component remained attached to the magnetic bead, which could be observed by a lack of change in pH as indicated by phenol red.

### Assessment of cleavage activity of 39E with fluorophore-labeled substrate

**Figure 2:**
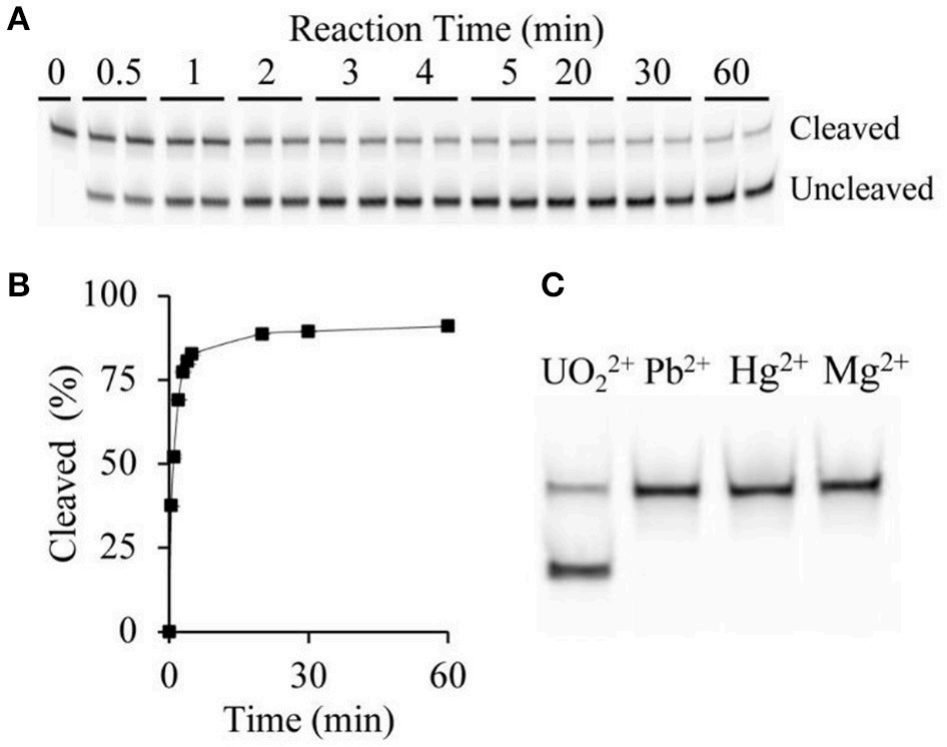
DNAzyme sensitivity and specificity. **(A)** Cleavage pattern showing uncleaved and cleaved bands following dPAGE of samples following uranyl-induced cleavage for varying times at 15 μg/L, with two replicates shown for each time point. **(B)** Time-course of uranyl-induced cleavage. **(C)** Comparing cleavage after 5 min incubation with 15 μg/L uranyl, alongside 10 μg/L lead ions, 6 μg/L mercury ions, and 200 μg/L magnesium ions.

In an effort to develop a biosensor capable of rapid detection of uranyl, initial investigations focused on the percentage of observed cleavage as a function of time (Figure 2A). Since cleavage led to release of the 3′ portion of the substrate, we were able to assess cleavage efficiency by using a substrate sequence with a covalently linked 5′ terminal fluorophore (6-FAM) to track cleavage. The FAM-labeled fragment of the cleaved substrate migrates faster than the uncleaved substrate on denaturing polyacrylamide gel electrophoresis (dPAGE) (Figure 2A) and therefore uranyl-induced cleavage was observed. The cleavage reaction was quenched after time points ranging from 30 s to 60 min. Increasing percent cleavage was observed with increasing time, however the reaction reached a plateau at approximately 5 min, thus this was set as the incubation time (Figure 2B).

### Specificity of the 39E-UrDNA complex

The extension of the terminal ends of the substrate, which hybridizes with the DNAzyme to form the two duplex regions flanking the cleavage site DNAzyme-substrate complex (Figure 1), could potentially alter the specificity of the active DNAzyme domain. Therefore, we set out to determine if the specificity of the 39E-UrDNA system was affected by evaluating the modified DNAzyme's specificity to Pb^2+^, Hg^2+^, and Mg^2+^, which were chosen to represent potential common metal ion interferents. Polyacrylamide gel electrophoresis was used to assess the cleavage activity of 39E-UrDNA in the presence of these metals (Figure 2C). After 5 min, the percent cleavage of the 39E-UrDNA was 80.9 ± 1.5%, whereas the percent cleavage of Hg^2+^, Pb^2+^, and Mg^2+^ were each no higher than background. The percent cleavage and specificity observed were consistent with the specificity demonstrated by the 39E DNAzyme as originally assessed by Liu et al. (2007).

It should be noted that the 39E DNAzyme, initially isolated by Liu et al. (2007), has been characterized extensively (Liu et al., 2007; Brown et al., 2009; Cepeda-Plaza et al., 2013). In their initial efforts, Liu et al. demonstrated the sensitivity of 39E by comparing its ability to generate a cleavage-induced increase in fluorescence in the presence of many competing metal ions (Liu et al., 2007). Brown et al. further characterized 39E by detailing its biochemical properties such as its pH dependent responsiveness, and identifying the conserved sequence required for catalysis (Brown et al., 2009). Higher resolution details of the binding regions of uranyl were later obtained by Cepeda-Plaza et al. (2013), using a uranyl-dependent photocleavage strategy to perform DNA foot-printing of the sequence (Cepeda-Plaza et al., 2013). Such detailed characterization studies were useful to us in the design of our biosensor which involved the coupling of additional components to existing DNAzymes, as they help establish regions which should not be altered in these designs.

### Colorimetric detection of uranyl in water using the modified litmus test

**Figure 3:**
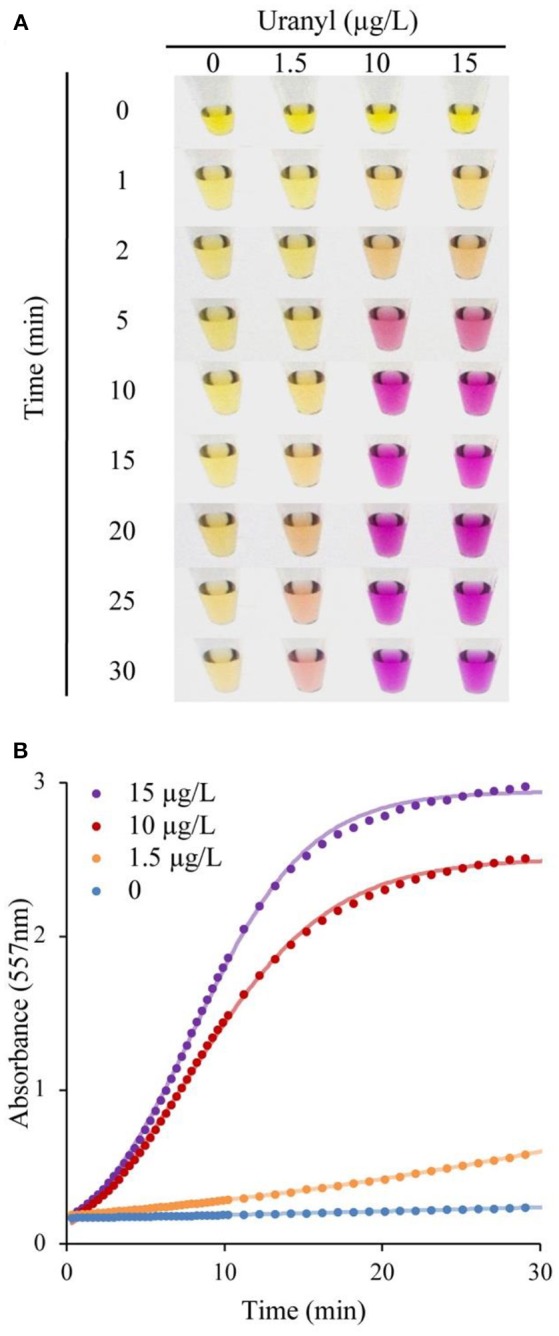
DNAzyme-induced colorimetric uranyl detection assay, using visual and spectrophotometer-assisted signal detection. **(A)** Colorimetric change for samples incubated with 0, 1.5, 10, and 15 μg/L uranyl, shown at 0–30 min. **(B)** Absorbance at 557 nm for 0, 1.5, 10, and 15 μg/L uranyl, over a 30 min period.

Following incubation with varying concentrations of uranyl ions, the UrDNA released into the solution due to uranyl-induced cleavage was isolated by addition of 150 μL ddH_2_O to the suspension, followed by separation of the supernatant from the magnetic beads using a magnetic rack. 20 μL of supernatant was transferred to another microcentrifuge tube, combined with 2.5 μL of 0.04% phenol red, and 25 μL of the urea substrate solution. Following a 5-min incubation with samples containing varying concentrations of uranyl, a vivid color change was evident within 30 min for samples containing 1.5, 10, 15 μg/L uranyl. Importantly, this vivid change is observable within 10 min for detection of 10 and 15 μg/L, which correspond to half the regulatory limits (Figure 3A). The distinction between samples was evident at earlier time points if assessments were made using UV-visible spectroscopy to measure the absorbance at 557 nm (Figure 3B).

**Figure 4:**
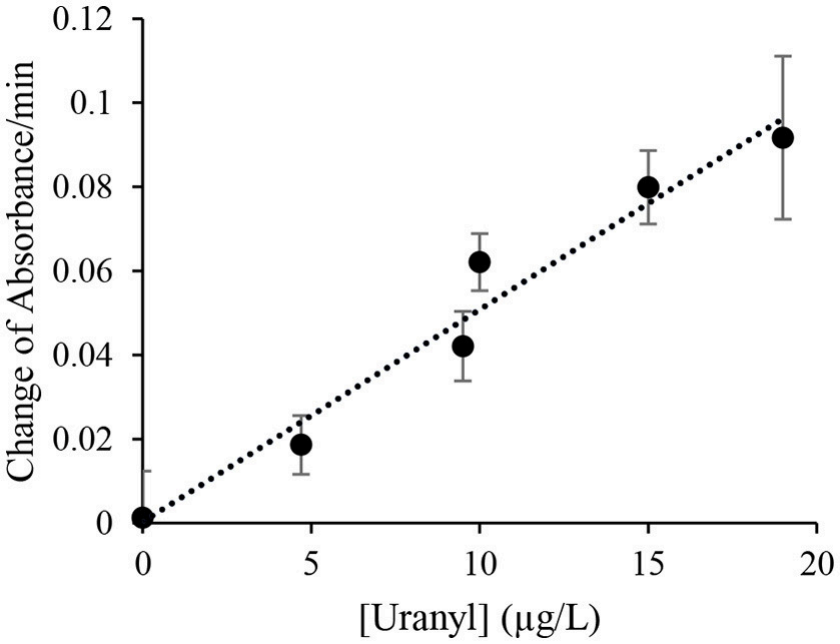
Initial signal rate as a function of uranyl concentration. The rate of increase in absorbance at 557 nm during the initial linear range at various uranyl concentrations is plotted as a function of the uranyl concentration in μg/L.

The initial response was linear, the signaling rate vs. the concentration of uranyl was then plotted as Figure 4 to obtain the limited of detection (LOD). By this method, the LOD was determined to be 0.052 μg/L (or 52 parts-per-trillion) based on the 3σ principle. However, spectrophotometry is not necessary for field applications of this biosensor as the color change produced by uranyl is distinguishable to the unaided eye after 10 min, at concentrations below the levels deemed safe for human consumption (Figure 3A).

### Detection of uranyl in environmental water samples using the 39E-UrDNA

**Figure 5:**
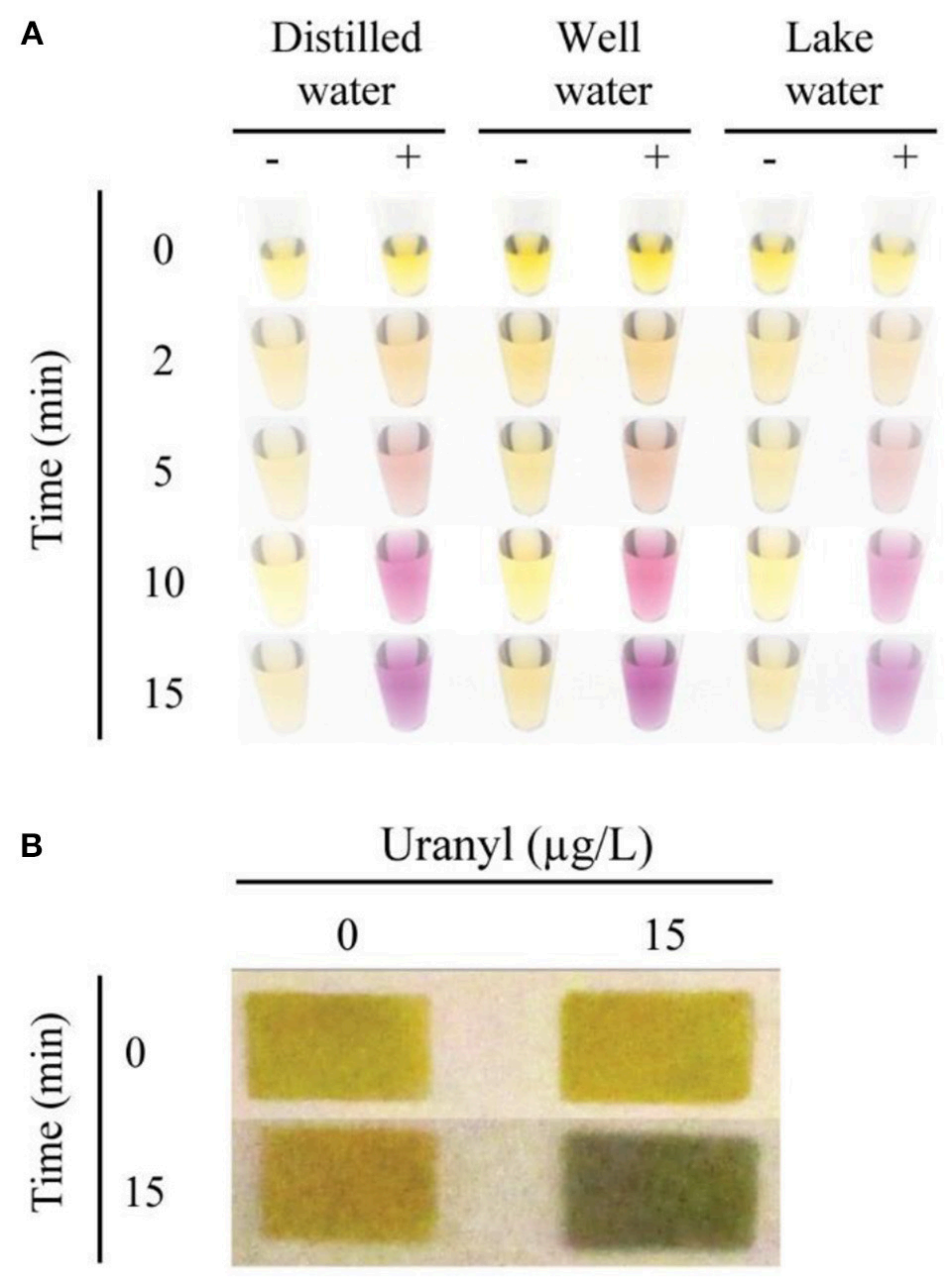
Colorimetric detection of uranyl in environmental water samples, using 39E-UrDNA. **(A)** Comparing the system at 0 and 15 μg/L uranyl, in distilled water, well water, and lake water at 0, 2, 5, 10, and 15 min. **(B)** Color change visualized using pH paper strips at 0 and 15 min, in well water samples spiked with 15 μg/L uranyl compared to a 0 μg/L control.

To test the potential application of this assay in environmental monitoring of water samples, we assessed the functionality of the 39E-UrDNA in response to uranyl spiked well water and lake water (Figure 5A). In this assay, distilled water (control), well water, and lake water were spiked with 15 μg/L uranyl. In each case, a vibrant color change from yellow to purple was observed all the samples containing 15 μg/L uranyl, while controls produced no discernable color change. The results in Figure 5A indicate that our sensing system can function without any hindrance in the presence of other contaminants found in environmental water samples, thereby presenting a viable alternative for use in the field and at home. Trace element analysis was performed on filtered and unfiltered samples of both lake and well water, with the results showing no detectable uranium and no differences between its detectable levels in unfiltered and filtered conditions (Table S1). The levels of a series of other elements were assessed to demonstrate the robustness of this assay in their presence (Table S1). While each complex matrix presents unique challenges, the trend observed herein aligns with previous results from our group for bacterial testing (Tram et al., 2014). To date we have demonstrated that this DNAzyme-based colorimetric biosensing approach produces a comparable response in both simple and complex matrices for targets differing in complexity from bacterial biomarkers to a metal ion target assessed herein.

### Paper-based detection of uranyl in spiked well water samples

The sensor response was also investigated using a paper platform as was described for the *E. coli* sensing DNAzyme-UrDNA system (Tram et al., 2014). UrDNA-based colorimetric biosensors investigated thus far all involve three general steps: cleavage by DNAzyme, hydrolysis by urease, and color change by pH indicator. In the solution-based approach, the final two steps occur in the same tube, as the phenol red is mixed with the urea solution. However, in the paper-based method, these steps are separated. Urease is allowed to hydrolyze urea in the absence of indicator dye in a brief incubation, prior to deposition on the pH paper. Whereas the color change of the indicator dye in the solution-based method is observable over the entire incubation period, in the paper-based method, the pH is measured after an incubation period of 15 min. As a result, we were able to demonstrate a simple paper-based alternative to the in-solution litmus assay presented using pH indicator dye (Figure 5B). In this assay, following uranyl-induced cleavage, the isolated supernatant was combined with the substrate solution containing urea, and allowed to incubate. After 15 min, the aliquot removed produced a distinct color change from yellow to green in the presence of 15 μg/L uranyl, while no color change was observed in the control (0 μg/L uranyl). Additional time-points and lower concentrations were also compared, as shown in the Supplemental Information.

Thus, we have demonstrated both solution-based and paper-based visualization strategies as viable approaches for monitoring the change in pH which occurs in the substrate solution following urease-catalyzed hydrolysis of urea.

### Advantages of the system and future improvements

One significant advantage of the featured approach is its simplicity, as it does not require either complicated equipment or highly trained personnel to conduct the test. This simplicity makes it suitable as a field test. Another key advantage is the modularity of the design, which allows for easy reconfiguration of the system into a new biosensor for detection of a different target with the use of a new DNAzyme that recognizes this target. In fact, many RNA-cleaving DNAzymes have been derived to specifically recognize diverse metal ions (McGhee et al., 2017), such as Pb^2+^ (Breaker and Joyce, 1994; Santoro and Joyce, 1997), Zn^2+^ (Li, 2000; Santoro et al., 2000), Mg^2+^ (Breaker and Joyce, 1995; Santoro and Joyce, 1997), Ca^2+^ (Faulhammer and Famulok, 1996; Zhou et al., 2016c), Na^+^ (Torabi et al., 2015), Hg^2+^ (Hollenstein et al., 2008), Cd^2+^ (Huang and Liu, 2015), Cr^3+^ (Huang et al., 2016), Ln^3+^ (Huang et al., 2014a,b, 2015), Ce^3+^ (Zhou et al., 2016a), and Ag^+^ (Saran et al., 2017). In theory, the design featured in this study can be reconfigured for these DNAzymes and any other future RNA-cleaving DNAzyme to be derived for a target of interest. This can be done simply through inclusion of a complementary sequence that can hybridize UrDNA in the substrate of a chosen DNAzyme.

A potential challenge for any simple biosensor to be used in a field test is to accommodate the variation in environmental temperature, which can vary significantly from region to region and season to season. Specifically for our test, the change in environmental temperature is expected to have some effect on the test based on the fact that most DNAzymes are selected to function under the temperature around 22°C (Breaker and Joyce, 1994, 1995; Geyer and Sen, 1997; Li, 2000; Silverman, 2005), and that urease has an optimal functional temperature of 60°C (Lai and Tabatabai, 1992). However, urease and most DNAzymes are still fairly active in the common environmental temperature range of 10–40°C (Lai and Tabatabai, 1992; Omar and Beauregard, 1995; Faulhammer and Famulok, 1996; Geyer and Sen, 1997; Fan et al., 2012; Li et al., 2012; Zhang et al., 2013; Zhou et al., 2015). Although our experiments were performed at the controlled room temperature of 22°C, we believe the test at other temperatures will produce comparable results. However, the speed of color change can be affected by the variation in environmental temperature, particularly when it is low, because both the DNAzyme and urease activities are lessened at the reduced temperature. Therefore, it is recommended that temperature-based calibration be carried out when the testing temperature significantly derivates from 22°C.

Another challenge associated with any biosensor that uses labile biological macromolecules as recognition elements is the loss of activity of such elements during the storage of the testing reagents. The use of urease and nucleic acids in the biosensor system makes this a relevant challenge. However, recently demonstrated methods that can stabilize proteins (Jahanshahi-Anbuhi et al., 2014, 2016) and nucleic acids (including RNA-cleaving DNAzymes) (Hsieh et al., 2017) through the use of a natural polysaccharide known as pullulan have been reported. The same approach can be used to solve the issue of reagent stability of our system.

A further improvement on our biosensor system is to enhance its operational simplicity by integrating the entire test into a simple device that can further simplify the testing procedure. For example, it is certainly very desirable to engineer a paper-based lateral flow device that only requires the addition of a test sample without the need for sample filtration, magnetic separation in one test tube and subsequent litmus test in another test tube that are used in our current testing procedure. This may be achieved through the creation of a two test-zone lateral flow device with a reaction zone that permits target-mediated DNAzyme cleavage and detection zone that reports the urease activity after lateral flow. Engineering such a device constitutes a focus in our future research effort.

## Conclusions

In this work, we describe a pH responsive biosensor that uses the 39E DNAzyme as a recognition element and urease as a mechanism to translate the cleavage event into a colorimetric response for the detection of uranyl in water. This simple and inexpensive assay enables detection of uranyl in environmental water samples at 15 μg/L in 20 min from start of the assay to finish. Since the change in color is concentration dependent, if higher concentrations of uranyl were present in environmental water samples, the test time would be significantly reduced. Additionally, the test can be further simplified by using commercially available pH paper which would decrease the number of reagents required. The presence of 15 μg/L uranyl in well water is indicated by a distinct color change of the pH paper from yellow to green, again with a complete assay time of 20 min. Both in solution and on paper, the concentration which can be detected is well below the maximum allowable concentrations set by regulatory bodies in Canada (20 μg/L), the US (30 μg/L), and globally by the WHO (30 μg/L). Uranyl is investigated as a target analyte in this assay in part due to the existence of the well characterized, and highly specific 39E DNAzyme, however the simplicity of this litmus-like assay can be easily adapted for use with other RCDs for the detection of other environmental contaminants.

## Author contributions

This work was conceptualized by SM, KT, and YL. Experiments were performed by SM, with the exception of trace element analyses, which were performed by JM. Experimental data were analyzed and figures were generated by SM and YL. The manuscript was prepared by SM, EM, and YL.

### Conflict of interest statement

The authors declare that the research was conducted in the absence of any commercial or financial relationships that could be construed as a potential conflict of interest.
