# Supplementary material for: Colorimetric Detection of Uranyl Using a Litmus Test
Source: Front Chem. 2018 Aug 9;6:332. doi: 10.3389/fchem.2018.00332 (PMC6095041; doi:10.3389/fchem.2018.00332)
Supplement: Supplementary file 1 [file Table_1.docx]

Supplementary Material

Colorimetric Detection of Uranyl Using a Litmus Test

Sepehr Manochehry, Erin M. McConnell, Kha Q. Tram, Joseph Macri, and Yingfu Li*

*** Correspondence:** Corresponding Author: liying@mcmaster.ca

# Supplementary Figures and Tables

## Supplementary Figure

0

Time (min)

0 1.5 10 15


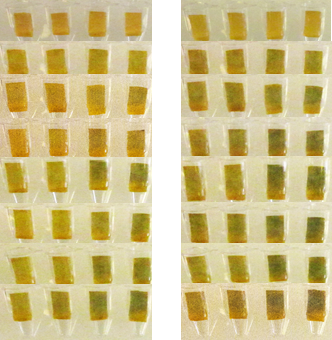


5

10

15

20

25

30

2

Uranyl (µg/L)

**Supplementary Figure 1.** Colorimetric detection of uranyl in well water samples, using 39E-UrDNA. Compared at 0, 1.5, 10, and 15 µg/L uranyl in well water after incubations of 0, 2, 5, 10, 15, 20, 25, and 30 minutes. pH strips are shown after being dipped at different time-points, into an aliquot of the supernatant combined with substrate solution.

## Supplementary Tables

**Supplementary Table 1.**

**Table S1.** Trace element analysis. Observed concentrations of each element shown, with less than symbols indicating values below a detection limit for respective method of detection. Comparing filtered and unfiltered well and lake water samples.

| Element | Unfiltered lake water | Filtered Lake water | Unfiltered well water | Filtered well water |
| --- | --- | --- | --- | --- |
| U | <0.010 nmol/L | <0.010 nmol/L | <0.010 nmol/L | <0.010 nmol/L |
| Cr | <1 nmol/L | <1 nmol/L | <1 nmol/L | <1 nmol/L |
| As | <0.01 µmol/L | <0.01 µmol/L | <0.01 µmol/L | <0.01 µmol/L |
| Pb | <1 nmol/L | <1 nmol/L | <1 nmol/L | <1 nmol/L |
| Co | <1 nmol/L | <1 nmol/L | 5 nmol/L | 5 nmol/L |
| Fe | <0.01 µmol/L | <0.01 µmol/L | <0.01 µmol/L | <0.01 µmol/L |
| Mn | <2 nmol/L | <2 nmol/L | 7.1 nmol/L | 8.1 nmol/L |
| Ni | 4 nmol/L | 3 nmol/L | 44 nmol/L | 45 nmol/L |
| Se | <0.01 µmol/L | <0.01 µmol/L | <0.01 µmol/L | <0.01 µmol/L |
| V | 8.6 nmol/L | 12.3 nmol/L | <1 nmol/L | <1 nmol/L |
| Sn | <1 nmol/L | <1 nmol/L | <1 nmol/L | <1 nmol/L |
| Cu | <0.01 µmol/L | <0.01 µmol/L | 0.08 µmol/L | 0.08 µmol/L |
| Zn | <0.1 µmol/L | <0.1 µmol/L | 7.1 µmol/L | 7.1 µmol/L |
| Al | <0.1 µmol/L | <0.1 µmol/L | <0.1 µmol/L | <0.1 µmol/L |
| Cd | <1 µmol/L | <1 µmol/L | <1 µmol/L | <1 µmol/L |
| Hg | <0.1 nmol/L | 0.3 nmol/L | 0.4 nmol/L | <0.1 nmol/L |
| Mg | 0.53 mmol/L | 0.51 mmol/L | 9.0 mmol/L | 8.8 mmol/L |
| HCO3 | <5 mmol/L | <5 mmol/L | <5 mmol/L | <5 mmol/L |
| Ca | 1.49 mmol/L | 1.50 mmol/L | 4.3 mmol/L | 4.3 mmol/L |
| Na | <20 mmol/L | <20 mmol/L | <20 mmol/L | <20 mmol/L |
| K | <1 mmol/L | <1 mmol/L | <1 mmol/L | <1 mmol/L |
| Cl | <20 mmol/L | <20 mmol/L | <20 mmol/L | <20 mmol/L |
